# Targeted analysis of nucleotide and copy number variation by exon capture in allotetraploid wheat genome

**DOI:** 10.1186/gb-2011-12-9-r88

**Published:** 2011-09-14

**Authors:** Cyrille Saintenac, Dayou Jiang, Eduard D Akhunov

**Affiliations:** 1 Throckmorton Plant Sciences Center, Kansas State University, Manhattan, KS 66506, USA

## Abstract

**Background:**

The ability of grass species to adapt to various habitats is attributed to the dynamic nature of their genomes, which have been shaped by multiple rounds of ancient and recent polyploidization. To gain a better understanding of the nature and extent of variation in functionally relevant regions of a polyploid genome, we developed a sequence capture assay to compare exonic sequences of allotetraploid wheat accessions.

**Results:**

A sequence capture assay was designed for the targeted re-sequencing of 3.5 Mb exon regions that surveyed a total of 3,497 genes from allotetraploid wheat. These data were used to describe SNPs, copy number variation and homoeologous sequence divergence in coding regions. A procedure for variant discovery in the polyploid genome was developed and experimentally validated. About 1% and 24% of discovered SNPs were loss-of-function and non-synonymous mutations, respectively. Under-representation of replacement mutations was identified in several groups of genes involved in translation and metabolism. Gene duplications were predominant in a cultivated wheat accession, while more gene deletions than duplications were identified in wild wheat.

**Conclusions:**

We demonstrate that, even though the level of sequence similarity between targeted polyploid genomes and capture baits can bias enrichment efficiency, exon capture is a powerful approach for variant discovery in polyploids. Our results suggest that allopolyploid wheat can accumulate new variation in coding regions at a high rate. This process has the potential to broaden functional diversity and generate new phenotypic variation that eventually can play a critical role in the origin of new adaptations and important agronomic traits.

## Background

Comparative analysis of grass genomes reveals a complex history and the dynamic nature of their evolution, which, to a large extent, has been shaped by ancient whole genome duplication (WGD) events followed by lineage-specific structural modifications [1]. In addition to ancient WGD, many lineages of grass species have undergone more recent genome duplications. It is hypothesized that WGD played an important role in the evolutionary success of angiosperms, providing opportunities for diversification of their gene repertoire [2]. Functional redundancy created by such duplication events can facilitate the origin of new gene functions through the processes of neo- and subfunctionalization. For example, evidence of ancestral function partitioning between ancient gene duplications was found in Poaceae [3,4]. In recent polyploids, transcriptional neo- and subfunctionalization [5,6] and tissue- and development-dependent regulation were demonstrated for duplicated genes [7-9]. These evolutionary processes can rapidly generate novel variation that allows for the diversification of grass species. The adaptive role of WGD is consistent with observations that, in the evolutionary history of many taxa, WGD often coincides with increased species richness and the evolution of novel adaptations [10,11].

Wheat is a recently domesticated, young allopolyploid species that originated in the Fertile Crescent. In addition to ancient WGD shared by all members of the Poaceae family [12], wheat has undergone two rounds of WGD in its recent evolutionary history. The first, hybridization of the diploid ancestors of the wheat A and B genomes, which radiated from their common ancestor about 2.7 million years ago, occurred 0.36 to 0.5 million years ago [13,14], resulting in the origin of the wild tetraploid wheat *Triticum dicoccoides *[15,16]. According to archeological records, the origin of domesticated tetraploid wheat, *Triticum turgidum *ssp. *dicoccum*, occurred about 8,000 years ago [17] and coincided with the origin of hexaploid bread wheat, *Triticum aestivum *(genome formula AABBDD). Domesticated forms of wheat demonstrate an incredible level of phenotypic diversity and the ability to adapt to various habitats. Even though the genetic basis of wheat adaptability is not completely understood, it most likely can be attributed to the plasticity of the polyploid genome [6,18].

The complexity and large size of the wheat genome (16 Gb for hexaploid wheat) has significantly delayed its detailed analysis. While recent studies have made progress in providing new insights into the dynamic nature of wheat genome evolution [19-24], analysis of molecular variation in coding sequences has received little attention. Comparative sequencing of a limited number of regions in the wheat genome revealed that some of the genes duplicated via polyploidy retained uninterrupted ORFs [21,25,26] whereas others were deleted or non-functionalized by transposon insertions or premature in-frame stop codon mutations [21,27]. Many of these mutations are associated with post-polyploidization events, which is suggestive of significant acceleration of evolutionary processes in the polyploid wheat genome [14,23]. To gain a better understanding of the global patterns of inter-genomic and intra-species coding sequence divergence and its impact on gene function, large-scale characterization of exonic sequences and gene copy number variation (CNV) in the wheat genome is required.

Although next-generation sequencing instruments are now capable of producing large quantities of data at low cost, complete genome sequencing of multiple individuals in species with large genomes is still too expensive and computationally challenging. In this vein, approaches have been developed that focus analysis on low copy non-repetitive targets. Such targets have been obtained by sequencing transcriptomes [28,29] or reduced representation genomic libraries [30,31]. Recently developed methods of sequence capture use long oligonucleotide baits for enrichment of shotgun genomic libraries with the sequences of interest [32-34]. These types of captures can be performed using solid- or liquid-phase hybridization assays [34,35]. Performance metrics of these two approaches have been shown to be quite similar [36]. However, the liquid-phase assay allows for a high level of multiplexing through the use of liquid-handling robotics. Integrated with next-generation sequencing, capture methodologies have shown high reproducibility and target specificity and have been effectively used for large-scale variant discovery in the human genome [37]. Fu *et al*. [38] presented the potential of array-based sequence capture in maize by discovering 2,500 high-quality SNPs between the reference accessions B73 and Mo17 in a 2.2-Mb region. More recently, the application of whole exome capture in soybean was used to identify CNV between individuals [39]. However, sequence capture has not yet been tested for the analysis of genetic variation in large polyploid genomes like that of wheat.

Here, we used a liquid-phase targeted exon re-sequencing approach to catalogue inter-genomic divergence, nucleotide sequence polymorphism, gene CNV and presence/absence polymorphisms (PAVs) between one cultivated and one wild tetraploid wheat accession. First, we evaluated the impact of polyploidy and intra-genomic gene duplications on the efficiency of variant discovery in the wheat genome by empirically validating identified variable sites. Using the overall depth of read coverage across genes and the depth of read coverage at variable sites, we were able to detect gene CNV resulting from gene deletions or duplications. Finally, we used the identified cases of gene CNV, gene sequence divergence and polymorphism to estimate the extent of genetic differentiation in coding regions between cultivated and wild tetraploid wheat, assess the potential impact of discovered mutations on gene function and biological pathways and gain a better understanding of evolutionary forces that shaped patterns of divergence and variation across the wheat genome.

## Results

### Specificity and uniformity of alignment

A total of 3.5 Mb of target sequence (3,497 cDNAs), represented by 134 kb of 5' UTR, 2,175 kb of coding and 1,160 kb of 3' UTR sequences, was captured from pooled samples from tetraploid wild emmer *T. dicoccoides *(Td) and cultivated durum wheat *T. durum *cv. Langdon (Ld) using liquid-phase hybridization and sequenced. Illumina reads were mapped to a reference prepared from full-length cDNA (FlcDNA) sequences. To increase the proportion of reads mappable to the cDNA reference, an additional data pre-processing step was incorporated to remove off-target intronic sequences. Introns were removed by iterating the alignment process and trimming unaligned reads by one nucleotide after each step, each time maintaining a minimal 30-bp read length.

After removal of intronic regions, homogeneity and depth of target coverage was significantly improved (Additional file 1). More than 60% of reads (383 Mb) were aligned to the reference sequence, which is 12% higher than that obtained for non-trimmed reads (Additional file 2). The median depth of coverage (MDC) increased to 13 reads per base, with 92% of targets covered by at least one read and 583 targets covered completely. Out of 3,497 FlcDNAs, 2,273 had a MDC of at least 10 reads per base. The MDC for the genomic regions included in the assay (GPC locus, 43 kb) was 19 for genic regions (5' UTR, exons, introns, 3' UTR). As the targeted genes represent about 0.035% of the tetraploid wheat genome, we achieved about 2,900-fold enrichment of the target sequences in the captured DNA.

In addition to reads that cannot be mapped to the cDNA reference in our experiment due to the presence of intronic sequences, previous studies showed that a significant fraction of unalignable reads can result from captures including off-target sequences or sequences that cannot be uniquely aligned to a genome [40]. In our study, the use of a genomic reference sequence from the GPC locus and the entire sequence of FlcDNAs (not just the 1,000 bp from the 3' end) resulted in a 1.4% (compared to the total number of aligned reads) increase in the number of reads mapped to the reference (5.5 Mb more), with the MDC progressively decreasing and reaching zero around 100 bp away from the target borders (Additional file 3). Moreover, around 7% (1.2 millions) of reads were not included in the alignment because of ambiguous mapping positions. Together, these data suggest that a significant portion of unaligned reads in our assay were due to the presence of hybrid (introns/exons or off-target/in-target) or non-unique reads.

**Figure 1:**
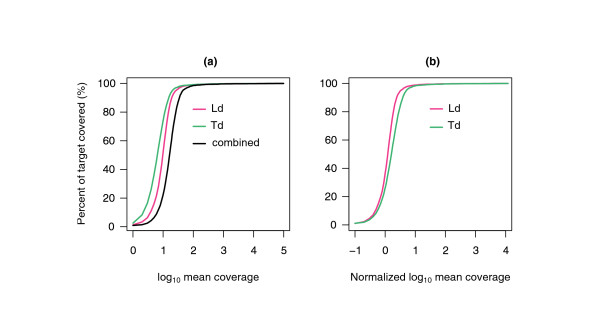
**Uniformity of cDNA target coverage**. **(a) **Proportion of cDNA targets covered by reads generated for Ld and Td genomes achieving mean target coverage (log10 transformed) equal to or greater than that indicated on the x-axis. **(b) **Proportion of cDNA targets with normalized mean coverage (log10 transformed) equal to or greater than that indicated on the x-axis.

Adaptor tagging sequences were used to separate reads generated from the Td and Ld libraries pooled together prior to sequence capture. The number of reads aligned to the reference sequences was 5.9 Mbp for Ld and 4.6 Mbp for Td, resulting in 3.1 Mbp (88%) of target sequence in Ld and 2.8 Mbp (79%) of target sequence in Td covered by at least one read (Additional file 2). Moreover, 65% of targets were covered by at least two reads in both wheat lines. The uniformity of target coverage obtained for Td and Ld was compared by plotting the cumulative distribution of non-normalized and normalized log10 mean coverage (Figure 1). The mean coverage was calculated for each individual cDNA target by dividing the coverage at each base by the total length of a cDNA target. The normalization was performed by dividing coverage at each base by the mean coverage per base across all targets. For targeted sequences we estimated the proportion of bases having coverage equal to or lower than the values indicated on the x-axis in Figure 1. The difference in coverage level between Ld and Td was mostly caused by the larger number of reads generated for Ld rather than sample-specific differences, thus suggesting that targets in both Ld and Td genomes were captured with a similar efficiency. These results are consistent with studies showing that variation in the depth of coverage among samples is not stochastic; rather, depth of coverage is mostly determined by the physicochemical properties of the baits [34]. Therefore, the pooling strategy applied in our study is an efficient approach for increasing the throughput of targeted re-sequencing experiments.

### Factors determining sequence capture assay efficiency in the wheat genome

Factors that govern the uniformity of coverage are critical to improving capture efficiency. The quality of a set of baits was assessed according to three parameters: consistency, sensitivity and complexity. Consistency relies on homogeneity of the set of baits in the capture assay, whereas sensitivity determines the bait's capacity to form secondary structure. Complexity refers to the abundance of a bait sequence in the capture sample. Bait GC content and melting temperature (T_m_) were calculated to assess the consistency of a pool of baits in the capture assay. The sensitivity of capture baits was estimated by calculating their minimum folding energy (PMFE), hybridization folding energy (PHFE), hairpin score and dimer score. The complexity of the assay was evaluated by comparing the frequency distribution of k-mers (k = 32) in targeted sequences with that of the entire wheat genome. Each of these parameters was compared with the MDC obtained for each of the 47,875 2× tiled baits (Additional file 4).

As expected, the bait GC content and melting temperatures T_m_1 and T_m_2 showed similar MDC distribution. Capture efficiency reached a maximum at 53% GC content, T_m_1 = 79°C and T_m_2 = 100°C (Additional file 4). Optimal coverage was observed for baits having a GC content ranging from 35% to 65%, which is in the same range reported previously for liquid-phase capture assay [34]. The hairpin score showed a weak effect on bait MDC compared to that of the dimer score, PHFE and PMFE (Additional file 4). The abundance of bait sequence in the wheat genome showed a strong positive correlation with target MDC, explaining 50% of observed MDC variation.

The presence of repetitive sequences in the capture assay resulted in non-homogeneous coverage of a small fraction of the target sequences. The observed MDC of 13 reads per base was significantly lower than the expected MDC (109 reads per base) estimated from the total number of reads and length of targeted sequences. The nature of highly abundant targets was determined by comparing target sequences with databases of known repetitive elements. A total of 87 FlcDNAs in the capture assay showed varying degrees of similarity to transposable elements (TEs) present in the databases (data not shown). The reads covering these targets represented about 37% of all generated reads. Apparently, the FlcDNA database TriFLDB contains cDNAs either originating from or containing insertions of TEs and other low complexity sequences, which resulted in a lowering of the expected target coverage. The frequency of sequences similar to the class II TE family (51%) was higher in the capture targets than that of sequences similar to the class I TE family (38%). Among repetitive targets showing similarity to TEs, no significant differences in the depth of coverage were observed between Ld and Td. A total of 21 high-coverage (maximum coverage > 500 reads) FlcDNA targets showed no hits to known TEs. Three of these targets corresponded to ribosomal protein genes, eight contained simple sequence repeats and five corresponded to multigene families. The remaining five targets may represent new TE families. Most of these repetitive targets contain k-mers highly abundant in the wheat genome, which demonstrates that the k-mer index is an efficient tool for filtering high-copy targets in complex genomes. Therefore, in addition to screening against the databases of known TEs, the usage of k-mer frequency screening to remove highly abundant targets in genomes should be considered for designing an optimized capture assay.

Two levels of target tiling, 1× and 2×, were compared to investigate the effect of tiling level on target capture efficiency. Different regions of the GPC locus were tiled with a set of non-overlapping (1× tiling) or overlapping baits. The 2× tiled targets showed higher depth of coverage compared to 1× tiled targets (Additional file 5). An MDC of 28.5 reads was obtained for 90% of the 1× tiled target bases whereas the MDC obtained for 2× tiled targets was 42.5 reads. Moreover, an increased level of tiling also resulted in more homogeneous target coverage (Additional file 5). However, even though 2× tiled targets were captured more efficiently than 1× tiled targets, the latter tiling strategy is more cost-efficient for targeting a large number of regions in a single capture reaction. By combining different parameters (thermodynamics of bait features, k-mer frequency index and tiling strategy) it is possible to optimize the design of a capture assay to efficiently target a large number of 'high-value' regions in the wheat genome.

### Genotype calling in the tetraploid wheat genome

**Figure 2:**
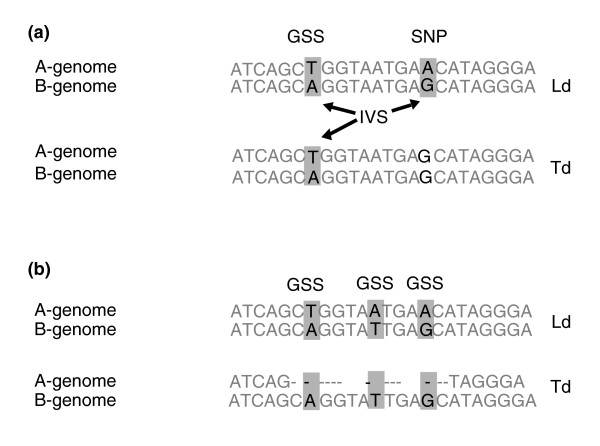
**Types of variable sites in the tetraploid wheat genome**. **(a) **At genome-specific sites (GSSs) nucleotide variants represent fixed mutations that differentiate the diploid ancestors of the wheat A and B genomes brought together by interspecies hybridization resulting in origin of allotetraploid wheat. SNP sites originate due to a mutation in one of the wheat genomes (in this example, in the A genome of Ld). Intra-species variable sites (IVSs) are highlighted in grey. **(b) **An example of CNV due to the deletion of a homoeologous copy of a gene. Deletion of a gene in the A genome of Td resulted in the disappearance of three bases, T, A and A, in the alignment.

Short read sequencing technologies are less suitable for reconstructing haplotypes of each individual wheat genome. In our alignments, Illumina reads from homoeologous or paralogous copies of a gene can be mapped to the same region of the reference sequence. Thus, the primary challenge for variant discovery in these complex alignments was distinguishing allelic variation between lines (henceforth, SNPs) from sequence divergence between the wheat genomes (henceforth, genome-specific sites (GSSs)) (Figure 2a). If only one polyploid wheat line is considered, a variable site cannot be classified as a GSS or SNP until it is compared with the sequence of the same genomic region from another wheat line. For that reason we defined sites with two nucleotide variants within a single wheat line as intra-species variable sites (IVSs). Then, according to our definition, GSSs should have IVSs present in both Ld and Td, whereas the characteristic features of SNP sites will be the presence of an IVS in one of the two wheat lines (A and G in Figure 2a) and a monomorphism for one of the variants in another line (G in Figure 2a). Patterns of variation in polyploid alignments are further complicated by intra-genomic gene duplications due to paralog-specific mutations accumulated in duplicated genes (excluding genes duplicated via polyploidization).

One of the possible sources of errors in genotype calling in polyploid alignments is failure to sequence one of the variants at an IVS. We estimated the theoretically expected probability of not recovering both variants at an IVS due to chance alone by assuming equal frequencies of each variant in a sample of sequence reads. If coverage depth at a particular IVS is Poisson distributed with parameter *λ*, the probability of sequencing only one of the two variants is *p *(one variant | *λ*) = 2exp(-*λ*). Then, the probability of obtaining *T *sites where we failed to recover a second variant in the Td and Ld genomes can be approximately calculated using the formula:

p(T)=2×p(one variant|λ)×t

where *t *= 0.02 × 3.5 × 10^6 ^is the expected number of mutations in all target sequences assuming 2% divergence between the wheat genomes in coding regions [26]. Using the experimentally obtained mean read coverage (*λ *= 13) for single copy targets, the estimate of *T *is 0.3 false positive variants in 3.5 × 10^6 ^bp of target sequence.

In order to identify SNPs and reduce the number of false positives after genotype calling, we applied several post-processing filters. Filtering parameters were determined by analyzing Sanger re-sequencing data obtained for a subset of gene loci targeted by the capture assay. The following filtering steps were used. First, variable sites present in genes showing unusually high depth of coverage were excluded due to possible alignment of duplicated copies of genes or repetitive elements. The cut-off MDC value was based on the 99th percentile of MDC distribution calculated for gene targets that showed similarity to single copy wheat ESTs mapped to the wheat deletion bins [41]. Out of 3,497 genes, 57 with a MDC higher than or equal to 61× (the cutoff MDC value) were filtered out. Second, a minimum coverage threshold of eight reads per base was applied to call a site monomorphic in one of the wheat lines when another line had an IVS (SNP site according to Figure 2a). Third, an experimentally defined threshold was applied to the ratio of variant coverage at an IVS calculated as the log2 ratio of the number of reads covering one variant relative to that of another variant. This filter was used to remove IVSs due to the alignment of paralogous copies of genes and was based on the following assumptions: the ratio of variant coverage at an IVS for single-copy genes assuming equal efficiency of capturing A and B genome targets is similar; and alignment of paralogous sequences will produce a coverage ratio deviating from the expected 1:1 ratio. However, due to variation in probe capture efficiency and stringency of alignment, we expected some deviation from a 1:1 coverage ratio even for single-copy genes and empirically estimated upper and lower thresholds of variant coverage at an IVS in a selected set of single-copy genes (described below). IVSs producing a coverage ratio outside of this estimated range were discarded.

**Figure 3:**
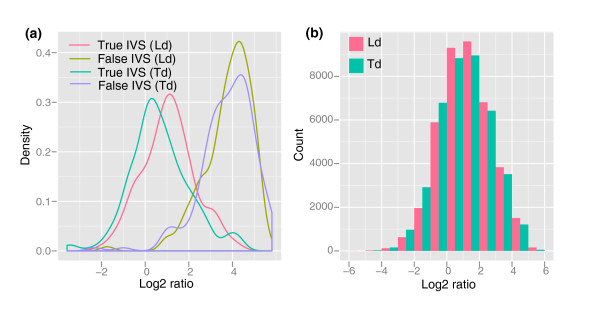
**Ratio of read coverage at intra-species variable sites**. **(a) **Density distributions of log2 ratio of read coverage at IVSs. The log2 ratio of the coverage depth was calculated by dividing the number of reads harboring a variant similar to the reference sequence by the number of reads harboring an alternative variant. True and false IVSs correspond to variable sites confirmed or non-confirmed, respectively, by Sanger sequencing. **(b) **The distribution of log2 coverage ratio at all variable sites detected by mapping sequence capture data to the reference sequence.

To determine the confidence intervals of variant coverage deviation at IVSs, we calculated the distribution of coverage depth log2 ratio in a set of 20 randomly selected single-copy genes. Only those variable sites that have at least one read representing each variant in Ld and/or Td were included. According to genotype calling in sequence capture alignments, these 20 genes contained 286 and 309 variable sites in Ld and Td, respectively. Sanger sequencing recovered only 132 IVSs in Ld and 131 in Td (true IVSs), whereas the remaining sites turned out to be monomorphic (false IVSs). One of the most likely explanations for the presence of false IVSs is the alignment of diverged paralogous copies of genes. For each of the true and false IVS datasets, we calculated the log2 ratio of the coverage depth for a variant that matched the reference nucleotide base to the number of reads matching the alternative variant (Figure 3a). The log2 ratio distributions showed a very clear difference with a peak around 1 for true IVSs and a peak around 4 for other variable sites, suggesting that the log2 variant coverage ratio can effectively discriminate these two types of variation. The upper log2 ratio thresholds for true IVSs were set to 1.6 and 1.0 for Ld and Td, respectively. These values of log2 ratio should maintain the false IVS discovery rate below 5%, which is defined as the proportion of sites that appear as IVSs in sequence capture data but fail validation by Sanger re-sequencing.

The log2 ratio distribution at true IVSs also demonstrated that the wheat capture assay was capable of capturing diverged copies of genes from different wheat genomes with some bias toward the reference copy of a gene used for bait design. For example, the log2 ratios for Ld and Td suggest that the reference sequence bases have higher coverage than alternative variants. The same trend was observed for the log2 ratio calculated for the entire dataset (Figure 3b). Apparently heterogeneity observed in the efficiency of capturing sequences from different wheat genomes is explained by variation in the level of their divergence from a reference. Therefore, we should expect that genes or regions of genes highly diverged from a reference sequence will be captured less efficiently than genes showing high similarity to a reference.

The total length of target sequences having sufficient coverage for variant detection was about 2.2 Mb, within which, after applying filtering criteria to variation calls, we identified 4,386 SNPs, 14,499 GSSs (Additional file 6) and 129 small scale indels (Additional file 7). Discovered SNPs and GSSs were validated by comparing sequence capture data with Sanger re-sequencing data. Among 40 genes, 283 and 97 GSSs were identified by Sanger sequencing and sequence capture, respectively (Additional file 8). A total of 96 GSSs were shared between these two datasets, suggesting only a 1% (1 of 97) false positive rate but a nearly 66% false negative rate (186 of 283). Most of the false negative GSSs were due to low target coverage resulting in failure to recover a second variant at GSSs. Thirty SNPs were shared between the sets of 58 SNPs detected by Sanger sequencing and 43 SNPs detected by sequence capture, suggesting that the experimentally validated SNP false positive rate should be around 30% (14 of 43) with a 62% (17 of 27) false negative rate. In 12 cases, false SNPs were due to a failure to recover a second variant at a GSS and in 2 cases the false positives were due to the alignment of paralogous sequences. The fact that the theoretically expected impact (see above) of failure to sequence both variants at IVSs on the false positive rate is negligibly small suggests that other factors are involved in defining the false SNP discovery rate in the capture data.

Another factor that can impact the probability of recovering a second variant at IVSs is a high level of sequence divergence between the reference and captured DNA. To further investigate this source of error, we performed a BLASTN search of raw sequence data using 40-bp sequence fragments flanking false positive SNP sites. We found that 50% of the time we were able to recover reads harboring a second IVS variant that we otherwise failed to align to the reference sequence because the number of mutations differentiating these reads from the reference exceeded the threshold used for alignment. To reduce the overall SNP false positive rate below 30%, we applied this strategy for filtering all SNP sites. The resulting data consisted of 3,487 SNPs with an expected 15% false positive rate. When the GSS and SNP density per bait was compared with the median read coverage of targeted regions we observed that the depth of coverage decreases with increasing number of mismatches (Additional file 9).

### Copy number and presence/absence variation

Two different approaches were used to identify CNV and PAV in the Ld and Td genomes. To reduce variation due to inclusion of targets with low and/or non-uniform coverage, only those genes that had at least 70% of their sequence covered by at least one read were selected. The genes satisfying these selection criteria represented 75% (2,611) of all targets in the wheat capture assay.

#### CNV detection based on the level of target coverage

The CNV-seq method based on the relative depth of target coverage in Ld and Td detected 85 CNV targets (Additional file 10). To understand the molecular basis of these CNVs, we estimated the number of variable sites in each CNV target and compared it with the average number of variable sites per non-CNV target. We assumed that if a CNV target has no variable sites, the most likely cause of CNV is gene deletion in one of the wheat genomes. However, if a CNV target possesses variable sites, the cause of the observed CNV is the increased/decreased number of gene copies in a multigene family in one of the compared wheat lineages. In our dataset, the increased frequency of variable sites in CNV targets was suggestive of variation in gene copy number in multigene families. While the average number of variable sites for non-CNV targets in Td and Ld was 25 and 27, respectively, we found that for CNV targets, 41 variable sites in Td and 42 variable sites in Ld were present on average. Therefore, we concluded that among the detected CNV, 77 variants were due to an elevated number of target copies in the Ld genome and 8 variants resulted from copy increase in the Td genome. Among these gene families we found seven genes encoding proteins involved in response to biotic and abiotic stresses, eight genes encoding proteins regulating gene expression or translation, three kinase-encoding genes and twelve genes encoding proteins involved in cellular metabolism (Additional file 10).

Furthermore, we used the level of target coverage to identify cases of PAV. For this purpose we searched for targets that showed zero MDC in one of the wheat lineages and a MDC of at least 10 reads in another lineage. Four complete gene deletions in Td and one complete gene deletion in Ld were detected and positively validated by PCR (Additional file 11).

#### CNV detection based on variant coverage at IVSs

The variant coverage data at IVSs were also used to detect cases of gene deletion in one of the homoeologous chromosomes. The characteristic feature of these deletions is the presence of a single variant in one of the two wheat lines and both variants in another one. Although these types of sites can be valid SNPs (Figure 2a), a high density per gene target may signify that this site is the consequence of complete or partial gene deletion in one of the wheat genomes (Figure 2b). Therefore, all gene targets bearing more than 70% of variable sites represented in one of the two wheat lines by only one variant were classified as gene deletions. Nine cases suggesting a deletion of one of the two homoeologous copies of genes were discovered in our dataset (Additional file 11), with eight deletions found in Td and one in Ld. All deleted gene loci were partially re-sequenced by the Sanger method and eight deletion events were positively validated. Four genes (contigs 1469, 1938, 3750, and 3935) showed a complete deletion of one homoeologous copy whereas contig4241 carried only a partial deletion. Contigs 3780 and 4476 showed evidence of reciprocal deletion of one of the homoeologous copies of a gene; in this case Ld and Td each contained a gene copy from different wheat genomes.

### Patterns of variation and divergence in wheat genomes

The GSS and SNP data were used to assess the impact of polyploidization on gene evolution and the extent of divergence between cultivated and wild wheat lineages. Previous analyses of GSSs in the polyploid wheat genome did not detect evidence of inter-genomic gene conversion and/or recombination, which was arguably attributed to the effect of the *Ph1 *gene [42]. Therefore, since most GSSs correspond to sites of divergence between the wheat genomes inherited from the diploid ancestors, they can be used to ascertain evolutionary processes at the diploid level. Although there is a small probability for some GSSs to be SNPs whose coalescence time predates the divergence of the cultivated and wild tetraploid wheat lineages, the proportion of these polymorphic sites relative to divergent mutations between the diploid ancestors is expected to be negligibly small. This is supported by the fact that in the diverse population of wild emmer, the average number of pairwise differences per site among gene sequences (*π *≈ 10^-3^) [43] was 200 to 500 times (2 to 5 × 10^-2^) lower than the divergence between the wheat genomes [26]. We took advantage of having sequences of both wheat genomes to infer the ancestral and derived SNP allelic states using inter-genomic sequence comparison. For example, in Figure 2a the derived state corresponds to nucleotide 'A' and the ancestral state corresponds to nucleotide 'G'.

Out of 3,487 SNPs, 1,506 derived alleles were found in the Td lineage and 1,981 derived alleles were found in the Ld lineage, resulting in a density of derived mutations of 1.08 and 1.73 mutations per kilobase (SNPs/kb) in Td and Ld, respectively. The orientation of ancestral versus derived states was further validated by comparing SNP-harboring regions with EST sequences of diploid ancestors of the wheat genomes *Aegilops tauschii, Aegilops speltoides, Triticum urartu *and *Triticum monococcum *and othologous gene sequences from rice and *Brachypodium*. In most cases (85%) the orientation of the ancestral state inferred from inter-genomic comparisons was confirmed by comparison with outgroup species.

The density of derived SNPs in 5' (2 SNPs/kb) and 3' UTRs (1.6 SNPs/kb) was higher than in coding regions (1.3 SNPs/kb) in both the Ld and Td genomes (Additional file 12). Using the deletion bin mapped wheat ESTs [41], we assigned 518 genes to chromosomal regions (Additional file 13). These genes contained 2,233 GSSs, and 275 and 195 derived SNPs in Ld and Td genomes, respectively. We tested the relationship between the distance of the chromosomal region from the centromere and the density of GSS and SNP sites. Consistent with previous studies in other species [37,44], the density of divergent mutations (Pearson correlation *r*^2 ^= 0.32) and polymorphic sites in the Ld (Pearson correlation *r*^2 ^= 0.52) and Td (Pearson correlation *r*^2 ^= 0.58) genomes increased with increasing physical distance from the centromere (Additional file 13).

The impact of mutations on gene coding potential (Additional file 6) was assessed by mapping GSSs and SNPs to ORF annotations provided in the FlcDNA database. A total of 11,939 variations were identified in gene coding regions, leading to mostly synonymous changes as expected (Table 1). The genomes of cultivated and wild wheat were different from each other by 875 protein coding changes, of which 56% were found in cultivated wheat. The number of synonymous or non-synonymous SNPs relative to the total number of SNPs did not show a statistically significant difference between Ld and Td according to the Fisher exact test (*P *= 0.83 for non-synonymous SNPs and *P *= 0.77 for synonymous SNPs). Out of 20 loss-of-function (LOF) SNPs, a lower fraction was found in the genome of cultivated wheat. In addition, we identified seven cases of reverse mutations resulting in restoration of the ORF, five of which were detected in the Ld genome, and two of which were discovered in the Td genome. Since these reverse mutations may increase the length of the coding sequence, they may have a strong impact on gene function (Additional file 6). Comparison with the sequences of orthologous genes in *Brachypodium*, rice, *Ae. tauschii, Ae. speltoides, T. monococcum, T. urartu *and hexaploid wheat confirmed that the ancestral state corresponds to a stop codon. To exclude the possibility of annotation artifacts, the ORFs of each gene with reverse mutations were validated individually through comparison with the protein sequences in the NCBI database. In one case, a mis-annotated ORF was uncovered.

**Table 1:** Classification of genome-specific sites and SNP sites

Variable sites	Type of mutation	Count
GSS	Non-synonymous	2,925
	Synonymous	6,850
	Premature stop codons	26
Derived SNPs in Ld genome	Non-synonymous	485
	Synonymous	729
	Premature stop codons	7
	Stop codon loss	5
Derived SNPs in Td genome	Non-synonymous	363
	Synonymous	524
	Premature stop codons	13
	Stop codon loss	2

Groups of genes involved in processes important for local adaptation or selected during domestication may have patterns of variation at non-synonymous sites different from that of neutral genes. We investigated the enrichment of non-synonymous and synonymous SNPs and GSSs among genes grouped according to their biological function. For this purpose, all genes included in the wheat capture were classified into functional categories using the Blast2GO annotation tool and plants Gene Ontology (GO) terms (Additional file 14). A Fisher exact test with multiple test correction (false discovery rate (FDR) < 0.05) was used to compare the frequency of non-synonymous relative to synonymous mutations in different GO groups. This analysis showed under-representation of non-synonymous GSSs in genes involved in basic house-keeping biological processes related to cell metabolism (Table 2). Since, most of the GSSs are inherited from diploid ancestors, the data suggest that these categories of genes were preferentially subjected to purifying selection in the diploid ancestors of the wheat A and B genomes. Comparison of the distribution of synonymous and non-synonymous SNPs in Ld showed an under-representation of non-synonymous SNPs in translation, membrane cell and structural molecular activity (Table 3) GO categories. In Td, non-synonymous SNPs compared to synonymous SNPs were over-represented in genes involved in signaling, regulation of cellular processes, signal transmission and transduction and biological regulation (Table 3).

**Table 2:** Enrichment of Gene Ontology terms for genes with non-synonymous genome-specific sites

GO group	GO term	Name	FDR	Genes with non-synonymous mutations
Cellular localization	0009987	Cellular process	0.010	Under-represented
Molecular function	0003824	Catalytic activity	0.040	Under-represented
Biological process	0006091	Generation of precursor metabolites and energy	0.040	Under-represented

**Table 3:** Enrichment of Gene Ontology terms for genes with non-synonymous SNPs

Wheat accession	GO group	GO term	Name	FDR	Genes with non-synonymous mutations
Ld	Biological process	0006412	Translation	0.004	Under-represented
	Cellular localization	0005840	Ribosome		Under-represented
		0016020	Membrane	0.020	Under-represented
		0005623	Cell	0.050	Under-represented
	Molecular function	0005198	Structural molecular activity	0.003	Under-represented
Td	Biological process	0009987	Cellular process	0.001	Under-represented
		0006629	Lipid metabolic process	0.047	Under-represented
		0006091	Generation of precursor metabolites and energy	0.038	Under-represented
	Cellular localization	0016020	Membrane	0.001	Under-represented
		0009579	Thylakoid	0.048	Under-represented
	Molecular function	0003824	Catalytic activity	0.022	Under-represented
		0003700	Transcription factor activity	0.045	Over-represented
		0016787	Hydrolase activity	0.013	Under-represented
		0008270	Zinc ion binding	0.015	Over-represented

## Discussion

The size of the wheat genome (10 Gb for tetraploid wheat and 16 Gb for hexaploid wheat) precludes the analysis of large numbers of samples by direct whole genome sequencing, even considering the increased throughput of the latest versions of next-generation sequencing instruments. Reduction of the complexity of the wheat genomic DNA sample by enriching it with valuable targets will allow us to analyze a large number of samples at a relatively low cost. Further reduction in the cost of sequencing and increased throughput can be achieved by using multiplexing adaptor sequences added during library preparation [45]. In this study, we successfully demonstrated that a liquid-phase sequence capture approach can be efficiently used for targeted enrichment in genomic libraries from polyploid wheat. Moreover, we were able to recover sequences from differentially tagged libraries that were combined into a single pool prior to hybridization with capture baits. The application of this approach to genome-wide association mapping and population genetics studies in wheat is now possible, but the level of multiplexing will be an important factor to explore.

Unlike assays created for other organisms, our design was based on the sequences of FlcDNA. Despite this fact, we recovered wheat exons even though the sequences of many baits were only partially complementary to genomic targets near exon-intron boundaries. The percentage of reads on target (60%) and the number of covered target bases (92%) obtained in our analysis are comparable with the results obtained in other studies using the same enrichment method [34,38-40]. Even if some difference was observed between the depth of read coverage in genomic regions (the GPC locus) and FlcDNA sequences, the application of an iterative alignment/truncation procedure to remove non-reference genomic regions was shown to be an efficient strategy for improving the uniformity and depth of target coverage. The optimization of bait design, which should include the selection of low copy targets in the wheat genome while considering their exon-intron structure, and the optimization of bait sequence composition can further improve the efficiency of cDNA-based capture assays. Overall, our results show that EST/cDNA sequences can provide useful information for designing successful capture experiments for species with less developed genomic resources.

Our results show that baits designed using only one of the homoeologous copies of a gene are capable of capturing diverged gene copies from the A and B genomes of tetraploid wheat. It should be feasible, therefore, to capture most of the duplicated genes in the polyploid wheat genome using a reduced set of probes designed using only a single 'diploid gene complement'. Moreover, since the radiation of many wild ancestors of wheat occurred within the time range of divergence of the wheat A and B genomes [13,14], this wheat exon capture assay, with appropriate precautions, can be used for capturing exons from the genomes of species closely related to wheat, many of which represent valuable sources of genes for agriculture. Bias toward more efficient capturing of targets similar to the reference sequence, which is consistent with the observed negative correlation between the captured DNA/bait sequence mismatches and target coverage, suggests that the enrichment of targets from the genomes of wheat relatives will be most efficient for sequences least diverged from the wheat genome. A similar observation showing negative correlation between the level of sequence divergence from a reference genome and the level of enrichment was made in maize [38]. The relative coverage at variable sites suggests that the previously estimated 2% coding sequence divergence between the wheat genomes [26] can result in about a two-fold reduction in target coverage, on average, when a SureSelect capture assay is used.

In spite of the complexity of the wheat genome, we were able to perform a reliable discovery of divergent (GSSs) and polymorphic (SNP) sites in the inter-genomic alignments. Experimental validation was used to estimate the SNP FDR as well as to develop filtering criteria for its control. The factors shown to increase the SNP FDR included a failure to recover a second variant at true IVSs and alignment of paralogous sequences creating false IVSs. According to theoretical expectations assuming equal probability of recovering each variant, the probability of missing a second variant at an IVS by chance in our dataset was negligibly small. Therefore, the most likely explanation for the failure to recover the second IVS variant was the high level of target divergence from the reference genome, which can either reduce the capture efficiency [38] or impact the ability of alignment programs to map reads to the reference sequence. Even though for most targets we were able to recover both copies of genes, we confirmed that some genes or regions of genes have an unexpectedly high level of divergence between the wheat A and B genomes, precluding them from aligning to the reference sequence. According to our data, this high inter-genomic divergence can explain most of the type I error rate (92%) in variant calls. Whereas decreasing the stringency of alignment would allow more divergent sequences to align, it would also increase the fraction of paralogous sequences aligned to the reference sequence, thereby introducing another factor that can inflate the false variant call rate. Performing variant discovery only in the regions of a genome with high coverage depth appears to be an efficient way of increasing the chance of recovering a second variant at some IVSs, which, however, comes at the cost of either deep sequencing or increasing the false negative rate. In the future, detailed analysis of the complete wheat genome and identification of highly diverged regions will help to improve the uniformity of homoeologous target capture, further reducing the FDR. The second source explaining the type I error rate (alignment of paralogs) was effectively eliminated by filtering based on variant coverage ratio. With the availability of the complete wheat genome sequence, alignment of paralogous sequences can be effectively controlled by excluding ambiguously mapped reads. Overall, even though some improvements are still required in terms of SNP calling procedures to reduce FDRs, sequence capture appears to be a powerful technique for the large-scale discovery of gene-associated SNPs in the wheat genome.

Two approaches to CNV detection used in our study resulted in different sets of genes, suggesting that each method captured different aspects of variation in our dataset. The results of validation by PCR and Sanger sequencing suggest that the identified CNVs are true structural variants. The coverage ratio calculated for each IVS was shown to be an effective method for identification of CNVs due to gene deletions in one of the wheat genomes. However, this method did not detect any gene duplications except known highly duplicated repetitive elements (data not shown). Large variation in the coverage ratio among targets most likely limits the power of this test to detect small changes in the variant coverage ratio when a duplication event involves only a small number of genes. Previous analyses of the wheat genome revealed high frequencies of inter-chromosomal and tandem duplications [21,23]. The number of CNVs detected in our study certainly underestimate their true frequency at the genome scale, most likely due to several factors, including our focus on low copy genes, the inability of short sequence reads to resolve near identical paralogs, the short length of targets interrogated by the capture assay spanning only exonic regions of individual genes, and the technical limitations of the enrichment method resulting in high variation in target coverage. Therefore, to analyze fine scale CNV and PAV more accurately, sequence capture can be coupled with comparative genomics hybridization using probes spanning large contiguous segments of the genome [46], which, however, requires the availability of a complete genome sequence.

The majority of CNVs we discovered were due to the increased number of gene copies in one of the two wheat accessions, with a higher frequency of gene duplications observed in the cultivated wheat form. Many genes showing evidence of CNV are involved in plant response to biotic and abiotic stresses, signal transduction and regulation of biological processes. Considering the importance of some of these gene classes in adaptation, it is possible that increased CNV provided a selective advantage under certain conditions. This is consistent with a finding that biotic stress response genes showed detectable CNV in *Arabidopsis *populations subjected to artificial selection [47].

These sequence capture data provide interesting insights into wheat genome evolution following polyploidization and have allowed us to assess the extent of gene space differentiation between the cultivated and wild tetraploid wheat accessions. The overall distribution of GSSs and SNPs across the wheat genome was consistent with the expectations of the neutral model of molecular evolution and the effect of selection on linked neutral variation [48], which predicts a positive correlation between divergence, polymorphism and recombination rate. In previous studies, the rate of recombination in wheat was shown to increase with increased distance from the centromere and correlate positively with the rates of gene deletions and duplications [19,49]. Therefore, the recombination rate in the wheat genome explains well not only the rates of structural evolution but also the distribution of sequence variation and divergence along chromosomes. Recent genome-wide sequencing projects in maize and human genomes also revealed a positive correlation between divergence, polymorphism and recombination rate, which was explained by relationships between the efficiency of selection and recombination [37,44].

The effect of selection on local variation was inferred by studying the distribution of SNPs in coding and non-coding regions of the wheat genome. Previously, diversity studies of diploid organisms showed decreased levels of polymorphism (by about 50%) in coding regions compared to that in non-coding sequences [37,50], consistent with the effect of selection. Interestingly, in the polyploid wheat genome we were able to detect a similar trend, suggesting that selection was not significantly diminished by WGD. This observation is consistent with previous studies based on sequencing only a small fraction of coding regions in the wheat genome [43,51]. Overall, our data suggest that a significant amount of functional redundancy was retained even after WGD, which is consistent with studies showing that wheat can accumulate a higher density of ethylmethane sulfonate (EMS)-induced mutations than diploid species [52] as well as withstand large scale chromosomal deletions [53,54]. Retention of duplicated genes suggests their importance for wheat adaptation and probably indicates that these genes have been favored by natural and/or human-driven selection.

We found that durum wheat harbors 24% more derived SNPs than wild emmer wheat. Among these derived SNP alleles, a lower number of LOF mutations was found in cultivated wheat than in wild emmer wheat. We cannot conclude, based on our data, whether this trend is common for cultivated wheat in general without large-scale re-sequencing of cultivated and wild populations. However, while LOF mutations in wild emmer populations can still be segregating polymorphisms, these types of mutation in cultivated wheat, if they elicit a strong deleterious effect, could be under strong negative selection. In such a case, we should expect that human-driven selection will reduce the frequency of LOF mutations in cultivated wheat.

We investigated the effect of non-synonymous GSSs and SNPs on various functional categories of genes. It was previously hypothesized that the rate of gene evolution is driven by selection acting not only on a single gene but on a set of genes linked by functional interactions in gene networks [55]. Within gene networks the rate of non-synonymous mutations in essential genes was shown to be lower than that in non-essential genes, usually linked to terminal nodes of a network [55]. Our finding that non-synonymous divergent GSSs in polyploid wheat are under-represented in genes involved in the generation of precursor metabolites, one of the central components of a cell metabolic network, supports this hypothesis and suggests that this group of genes has been under purifying selection in the diploid ancestors of wheat genomes.

Analysis of derived SNPs showed under-representation of non-synonymous mutations in wild emmer wheat in the same functional category found for GSSs, generation of precursor metabolites, which might be indicative of selection acting to reduce amino acid changes in this functionally important group of genes. In cultivated durum wheat, under-representation of genes with non-synonymous SNPs was found only for a biological process related to translation. Similar under-representation of major-effect non-synonymous mutations in genes involved in translation was observed in *Arabidopsis *[50]. Although this result could be the consequence of neutral stochastic processes acting on segregating polymorphisms in the population, the fact that cultivated wheat is undoubtedly subjected to strong selection pressure is suggestive more of purifying selection acting to reduce non-synonymous changes in this group of genes. We found two GO categories of genes involved in transcription factor activity and zinc ion binding that showed accumulation of SNPs at non-synonymous sites. Since non-synonymous mutations in transcription factor genes may affect the ability of transcription factors to bind to regulatory elements, this evolutionary process has the potential to impact a large number of regulated genes and generate new functional variation.

Our study discovered a significant level of divergence in the coding sequence and gene copy number between the cultivated and wild wheat genomes. By extrapolating our estimates of non-synonymous and LOF mutations to the whole tetraploid wheat genome, assuming that it encodes 50,000 duplicated pairs of genes with an average length of 2,000 bp [23], and by correcting for experimentally defined error rates, we can predict that the genomes of wild and cultivated tetraploid wheat are distinguished from each other by nearly 68,000 amino acid changes and 1,000 LOF mutations. This level of divergence (0.7/gene) when the number of non-synonymous SNPs is normalized by the total number of genes in the wheat genome is higher than that reported for two human individuals (0.3/gene) [56] or *Arabidopsis *accessions (0.1/gene) [50] and most likely results from processes linked with polyploidization.

## Conclusions

Here, we show that exon capture, when combined with next-generation sequencing, is a powerful approach for targeted analysis of molecular variation in the complex wheat genome. Our study suggests a high level of differentiation in the coding regions of cultivated and wild tetraploid wheat genomes; additionally, this observed differentiation appears to be consistent with the increased rate of evolutionary changes in polyploids. Inter-genomic divergence data indicate a historical selective constraint in the diploid ancestors of the two wheat genomes that acts on genes important to metabolic processes. The reduced level of polymorphism in un-translated regions of the wheat genome compared to that of translated regions suggests that the selective constraint on coding sequences was not significantly reduced by WGD; apparently, most homeologous genes in polyploid wheat retain their functionality. We hypothesize that the ability of allopolyploids to adapt to a broad range of environmental conditions stems not only from new interactions established between homoeologous copies of genes inherited from the diploid ancestors but also from exploiting new functional variation generated at an increased rate.

## Materials and methods

### Capture assay design

Sequence capture in polyploid wheat was performed using Agilent's SureSelect solution phase hybridization assay. A total of 55,000 120-mer RNA baits were designed to target 3.5 Mb of sequence selected from 3,497 genome-wide distributed wheat FlcDNAs (Additional file 14) from the Triticeae Full-Length CDS Database (TriFLDB) [57]. All FlcDNA sequences were compared with each other to select only one representative homoelogous copy for each gene. The baits were tiled with 60 bp overlap to cover up to 1,080 bp from the 3' end of each FlcDNA. Out of 3,497 FlcDNAs, 1,073 were covered entirely. The length of target sequence (part of the cDNA covered by capture baits) per cDNA was selected based on the previous estimates of genetic diversity in the populations of wheat landraces and wild emmer wheat (π≈ 0.001 or 1 SNP every 1,000 bp between any two given individuals in the population [43]) to increase the chance of detecting at least one SNP per cDNA target between Ld and Td. The proportion of the targeted 5' UTR, coding and 3' UTR sequences was 4%, 65% and 31%, respectively. In addition, 634 baits were designed to cover 12 non-repetitive genomic regions from the GPC locus of *T. diccocoides *carrying eight genes or pseudogenes (DQ871219) [58]. To test the effect of target tiling level on capture efficiency, both 1× and 2× tiling were applied to different parts of the GPC locus. Capture assay was hybridized with differentially barcoded genomic libraries prepared from DNA of wild emmer and cultivated durum wheat. Captured DNA was sequenced on the Illumina GAII instrument, generating 17.8 million 40-bp reads (712 Mb).

### Construction of genomic DNA libraries

Two accessions of tetraploid wheat where included in the sequence capture experiment: the wild emmer accession (*T. dicoccoides*, PI 428082-2 from Turkey) selected from the natural population grown at the putative site of wheat domestication in Turkey; and durum wheat cultivar Langdon (*T. turgidum *var *durum*) adapted to grow in the northern parts of the US. Genomic DNA isolated from the 3-week seedlings was used for library construction. DNA concentration was determined spectrophotometrically using a Nanodrop-1000 (Thermo Scientific, Pittsburgh, PA, USA). For each genotype, 3 μg of genomic DNA dissolved in 60 μl of deionized water was fragmented to an average size of 200 bp by 15 minutes of sonication on ice at maximum intensity (Virsonic 50, Virtis, Warminster, PA, USA). The following steps were performed according to the standard protocol of Agilent with slight modifications. Fragment end-repairing, A-tailed ligation, adapter's ligation and final PCR were performed using the NEBNext^® ^DNA Sample Prep Reagent kit. The average fragment size and molar concentration of the genomic libraries following sonication were estimated using Bioanalyser (Agilent). Fragment end-repairing was carried out by incubation of the reaction mix for 30 minutes at 20°C (100 μl reaction volume, 10 μl T4 DNA ligase buffer supplemented with 10 mM ATP, 4 μl dNTP, 5 μl T4 DNA polymerase, 1 μl Klenow enzyme and 5 μl T4 polynucleotide kinase). A-overhangs were added by incubating the library for 30 minutes at 37°C in a 50 μl final volume with 5 μl Klenow enzyme, 10 μl dATP and 3 μl Klenow exo (3'5' exo-). Samples were purified on QIAquick columns (Qiagen, Valencia, CA, USA) after each of these three steps. Adapter pools with different sequence tags (barcodes) were ligated to the wild emmer and durum wheat libraries. Ligation reactions were performed for 15 minutes at room temperature using 5 μl of DNA ligase in a 50 μl final volume. Samples were purified using MinElute columns (Qiagen). Size selection of 200- to 300-bp fragments was performed on a 2% agarose gel followed by elution of DNA using Qiaquick columns (Qiagen). Eluted DNA was amplified by 14 cycles of PCR in a 50-μl reaction mix containing 0.4 μM primer-A (CAAGCAGAAGACGGCATACGAGCTCTTCCGATCT), 0.4 μM primer-B (AATGATACGGCGACCACCGAGATCTACACTCTTTCCCTACACGACGCTCTTCCGATCT) and 25 μl Phusion High-Fidelity PCR Master Mix. Finally, PCR products were purified on QIAquick columns (Qiagen) and the quality of the libraries was assessed using Bioanalyser (Agilent). DNA concentration was determined using Nanodrop (Thermo Scientific). The concentration of the library was adjusted to 147 ng/μl.

### Hybridization and sequencing

Solution phase hybridization was performed according to Agilent's standard protocol. In a 200 μl dome cap PCR tube, 250 ng of each DNA library were pooled with blocker numbers 1, 2 and 3 (Agilent SureSelect Kit), denatured for 5 minutes at 95°C and incubated 5 minutes at 65°C. In parallel, the hybridization solution was prepared by mixing buffers 1, 2, 3 and 4 from the SureSelect kit while keeping the solution at 65°C. We then mixed 13 μl of hybridization solution, 7 μl of the library, 5 μl of pre-warmed (65°C) mix of SureSelect Oligo Capture Library, 1 μl of water and 1 μl of RNase block. A drop of mineral oil (Sigma, St. Louis, MO, USA) was added on the top of the reaction mix to prevent evaporation and the sample was incubated at 65°C for 24 hours in a GeneAmp PCR System 9700 thermocycler (Applied Biosystems, Carlsbad, CA, USA). The capture targets were then selected by pulling down the biotinylated bait/target with streptavidin-coated magnetic beads (Dyna M270 Streptavidin, Invitrogen, Carlsbad, CA, USA). The obtained capture solution was desalted using MinElute columns (Qiagen). Two separate 18-cycle PCR amplification steps were performed with 1 μl capture target, 2.5 μl Herculase II fusion DNA polymerase (Stratagene, Santa Clara, CA, USA), 0.625 mM dNTP, and 2.5 μl SureSelect GA PCR primers in a 50 μl final volume. PCR products were pooled and purified on QIAquick columns (Qiagen). The quality and concentration of the capture sample were assessed on a Bioanalyser prior to sequencing on the Illumina GAII instrument as single-end 40-bp reads.

### Raw data processing and alignment strategy

A total of 23 million 40-bp reads were generated and 17.8 million passed through the Illumina chastity filter (NCBI SRA database accession SRA039453). To avoid misclassifying Ld and Td reads, we filtered for high quality tag sequences with a phred33 quality score equal to or above 15 within the first four nucleotides. Reads were then grouped into six datasets according to their tag sequences. Tags used for the Ld sample were AT (5,039,822 reads), GAT (2,511,360 reads) and TGCT (2,044,603 reads), whereas tags used for the Td sample were CCAGT (530,580 reads), CCGACT (2,626,002 reads) and no-tag (4,655,217 reads). Before aligning the sequence reads to a reference, the sequence tags were trimmed off. The reference sequence for alignment was created by concatenating all FlcDNA and GPC locus sequences.

Reads were aligned to reference sequences using bowtie-0.12.5 [59] with parameters -m1 and -n2 in order to, respectively, suppress all the reads with more than one reported alignment and permit two mismatches between the reference sequence and the first 28 nucleotides of a read. To increase the number of reads aligned to reference exonic sequences and improve homogeneity of coverage, non-aligned reads were trimmed from their 5' or 3' ends in order to remove intronic sequences. Briefly, bowtie was run with parameter -un to obtain non-aligned reads, which were then truncated by one base from the 3' or 5' ends and re-aligned. The minimum read length was maintained at 30 bp to reduce alignment of paralogous sequences. To account for differences in the length of reads after tag trimming, this process was performed separately for each of the six datasets. Mappable reads were pooled into three datasets, including Ld, Td, or Ld plus Td reads and aligned to the concatenated reference sequence.

Alignment files generated by bowtie were processed using SAMtools version 0.1.6 [60] to produce output in pileup format containing information about the depth of coverage and variant counts. All statistical analyses were performed using the R package. Python and Perl scripts used for processing alignment data are available from the authors upon request.

### Thermodynamics metrics and k-mer frequencies index

Only 2× tiled baits were selected for calculation of thermodynamic parameters. PHFE and hairpin and dimer scores were calculated using the python scripts provided by Xia *et al*. [61]. All scripts were run with default parameters except the PHFE script, which was run setting RNA as nucleic acid and temperature to 65°C. PMFE and melting temperature 1 (T_m_1) were calculated using metl.pl script [62] with the following parameters: -n RNA -t 65 and -N 1. A second method of melting temperature calculation (T_m_2) was implemented in the MELTING software [63], which was used with the following settings: -B RNA/DNA hybridization, -A sugimoto et al 1995, -N 1 and -P 6.15 × 10^14 ^(based on one million sequences in excess).

The frequency of k-mers in targeted sequences was compared with that of the whole wheat genome. Since a *k*-mer alphabet includes only four letters (A, T, C, G), it can be stored in *k *log2 4 = 2*k *bits. To maximally utilize the capacities of a 64-bit computer system and decrease computation time, we performed the indexing of the wheat genome using 32-mers. This value of k-mer may decrease k-mer resolution but can effectively capture unique k-mers [64]. K-mer counting was performed for the wheat genome shotgun sequence data [65]. All k-mers were enumerated and their values with associated frequency counts were stored in a MySQL database. A target sequence k-mer index was generated using the same approach and the frequency of their occurrence in the wheat genome was estimated. All the steps in this analysis were performed using Perl scripts.

### Variant discovery and copy number variation analysis

The alignments generated by bowtie were processed using SAMtools utilities. Variant calling was performed using the VarScan software [66] with default settings except the minimum depth of read coverage, which was set at two reads. Several post-calling filters were applied to the data to reduce the number of falsely identified variable sites. The filtering parameters are described in greater detail in the Results. Briefly, applied filtering included: 1) removal of variable sites showing unusually high depth of coverage to reduce the effect of repetitive sequences on variant calling error rate; 2) removal of variable sites showing an individual variant coverage ratio that significantly deviates from the expected 1:1 ratio (more details provided in Results); and 3) removal of variable sites that showed a level of coverage below specified thresholds. Selection of filtering parameters was based on Sanger re-sequencing of multiple gene fragments that were also targeted by the wheat sequence capture assay. To identify indels, gapped alignment was performed using BWA with default parameters [67]. The alignment files in BAM format were processed with Dindel [68] to extract the list of indels from the Ld and Td genomic alignments. Finally, we performed filtering step 1 as described above to eliminate indels present in highly abundant sequences.

Two approaches were used to identify genes showing evidence of CNV in Ld and Td. The first method of CNV detection relied on the ratio of target coverage in Td relative to Ld in a sliding window. The observed ratios were statistically assessed by estimating the probability of a random occurrence, given no CNV, using the method implemented in the CNV-seq software [69]. Only those targets that had at least four overlapping 500-bp windows (250-bp overlap) showing a statistically significant log2 coverage ratio were classified as CNVs. As a second approach, we utilized the depth of read coverage at variable sites to detect CNV assuming that gene deletion in one of the wheat genomes should be accompanied by reduced or absent coverage data for one or another variant in either the Ld or Td genomes. The gene targets that had at least 70% of their sequence covered by at least one read were selected for this CNV analysis.

For validation purposes, a total of 20 gene targets were re-sequenced using the Sanger method. Gene fragments were PCR amplified using exonic primers and amplicons were sequenced on an ABI3730xl instrument. Sequence alignment and variant discovery were performed using the Sequencher package (Gene Codes, Ann Arbor, MI, USA).

### Patterns of molecular variation

Annotation of FlcDNAs, including the 5' UTR, exon, and 3' UTR boundaries, were downloaded from TriFLDB [57]. Functional annotation of gene targets included in the wheat capture was performed using the BLAST2GO program (v.2.4.5) with default parameters [70]. Gene annotations were mapped to high-level broader parent terms, referred to as GO Slim terms, using the GO Slimmer tool [71]. The distribution of non-synonymous mutations among different functional categories of genes was compared with that of synonymous mutations using the Fisher exact test with multiple test correction as implemented in the BLAST2GO package.

The ancestral state at each SNP site was validated by comparing reference sequence with coding sequences of rice [72], *Brachypodium *[73], *Ae. speltoides, Ae. tauschii *and *T. monococcum *[6].

To estimate the distribution of FlcDNAs across the wheat genome, FlcDNA sequences were compared with deletion bin mapped ESTs [41] using the BLASTN program. Only hits with at least 97% similarity over 80 bp were considered. FlcDNAs with a significant hit to different ESTs were removed, as well as FlcDNAs with a significant hit to several ESTs mapped to different chromosomes. Chromosome arm positions for each mapped EST were defined by the middle of the deletion bin fraction length. If an EST was mapped to the same group of homeologous chromosomes, the deletion bin mid-points were averaged. TEs were annotated by comparing FlcDNA sequences with repetitive elements in the TREP [74] and RepBase databases [75] and the recently annotated set of TEs found by Choulet *et al*. [23]. The hits showing 80% similarity over at least 80 bp were considered significant. FlcDNA targets showing high depth of coverage but no significant hits to known TEs were analyzed individually for the presence of smaller TE fragments.

## Abbreviations

bp: base pair; CNV: copy number variation; EST: expressed sequence tag; FDR: false discovery rate; FlcDNA: full-length cDNA; GO: Gene Ontology; GSS: genome-specific site; IVS: intra-species variable site; Ld: *Triticum durum *cv. Langdon; LOF: loss-of-function; MDC: median depth of coverage; ORF: open reading frame; PAV: presence/absence variation; PHFE: probe hybridization folding energy; PMFE: probe minimum folding energy; SNP: single nucleotide polymorphism; Td: *Triticum dicoccoides*; TE: transposable element; UTR: untranslated region; WGD: whole genome duplication.

## Competing interests

The authors declare that they have no competing interests.

## Authors' contributions

CS designed the sequence capture assay, prepared enriched genomic libraries, performed bioinformatics and statistical analyses and participated in drafting the manuscript; DZ performed bioinformatics and statistical analyses; EA conceived the experiment, designed the sequence capture assay, performed bioinformatics and statistical analyses and drafted the manuscript. All authors read and approved the final manuscript.

## Supplementary Material

Additional file 1 **Depth of read coverage near exon junctions**. The improvement of depth of read coverage near exon junctions was obtained by iterative alignment and trimming unaligned reads by one nucleotide after each step. Depth of coverage along FlcDNA1028 obtained without read trimming (black) and with read trimming (red). Median depths of coverage for the gene obtained without read trimming or with read trimming are represented, respectively, by black and red horizontal lines. Click here for file

Additional file 2 **Capture assay statistics**. This file provides the details of alignment statistics (length of reads, total number of reads generated, total number of reads aligned after and before trimming, percentage of target covered) obtained by mapping captured reads to the reference sequence. Click here for file

Additional file 3 **Median depth of coverage per base around GPC locus target boundaries**. A set of 17 boundaries from the GPC locus having a depth of coverage less than 61× was selected. The average MDC was calculated for 17 off-target/on-target boundaries, including 500 bp upstream and downstream sequences. Click here for file

Additional file 4 **Influence of bait properties on capture efficiency**. MDC was calculated for 47,874 2× tiled baits. All baits with a MDC above 200 were removed from the analysis. MDC was plotted against different bait parameters values: GC, bait GC content; k-mers, median frequency of the bait sequence in the *Chinese spring *genome; PMFE, probe minimum folding energy; PHFE, probe hybridization free energy; HS, bait Hairpin score; DS, bait Dimer score; T_m_1 and T_m_2, melting temperatures 1 and 2. Details of parameter estimations are provided in the Materials and methods. Red curves represent the median of MDC per value of a parameter. Click here for file

Additional file 5 **Capture efficiency for 1× and 2× tiled regions of the GPC locus**. (a) The cumulative distribution of MDC for 1× tiled (black lines) and 2× tiled regions (red lines). **(b) **The MDC per base along the bait for 1× tiled (black lines) and 2× tiled targets (red lines). Click here for file

Additional file 6 **Impact of GSSs and SNPs on coding sequence**. Excel file showing the distribution of GSSs and SNPs between silent and replacement codon positions. Click here for file

Additional file 7 **List of discovered insertions and deletions**. Excel file containing indels identified in the Ld and Td genomes. Click here for file

Additional file 8 **Validation of SNPs and GSSs by Sanger re-sequencing**. Excel file containing a comparison of GSS and SNP sites between the sequence capture and Sanger sequencing datasets. Click here for file

Additional file 9 **Impact of mismatches between captured DNA and bait sequences on the median depth of coverage**. The number of mismatches (includes SNPs and GSSs discovered between Ld and Td) between the captured DNA and bait sequences were plotted against the median depth of target coverage obtained for a region covered by bait. Click here for file

Additional file 10 **Annotation of CNV genes**. Excel file listing CNV targets based on the CNV-seq analysis. Click here for file

Additional file 11 **FlcDNAs showing gene deletions in Ld or Td**. Excel file listing five cases of PAV based on MDC and nine cases of homoelog-specific gene deletions based on the absence of a second variant at IVSs. Click here for file

Additional file 12 **Density of GSSs and SNP in coding and non-coding regions**. Excel file showing the density of GSSs and SNP sites in 5' UTRs, exons and 3' UTRs. Click here for file

Additional file 13 **Distribution of SNPs along the wheat chromosomes**. Excel file showing the location of each FlcDNA in the wheat deletion bin map [41] determined by comparing FlcDNA sequences with the sequences of wheat deletion bin mapped ESTs using the BLASTN program. Click here for file

Additional file 14 **Annotation of targeted genes**. Excel file showing the annotation of genes included in the wheat sequence capture assay. Annotation was performed using the blast2GO program followed by assigning genes to functional groups defined by plant GO terms. Click here for file
