# Supplementary figures and images for: Targeted analysis of nucleotide and copy number variation by exon capture in allotetraploid wheat genome
Source: Genome Biol. 2011 Sep 14;12(9):R88. doi: 10.1186/gb-2011-12-9-r88 (PMC3308051; doi:10.1186/gb-2011-12-9-r88)

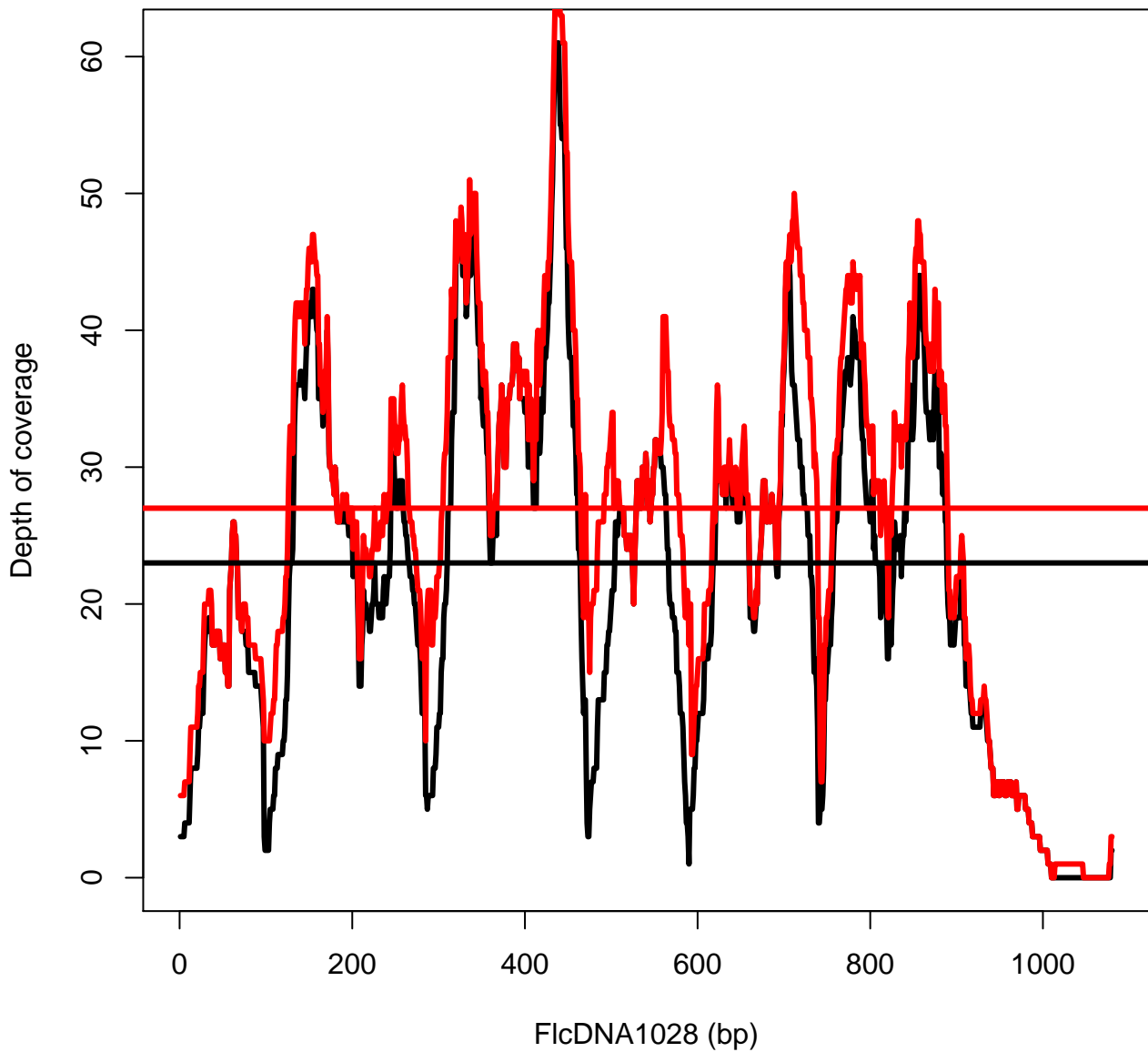

Supplement: Additional file 1 — Depth of read coverage near exon junctions. The improvement of depth of read coverage near exon junctions was obtained by iterative alignment and trimming unaligned reads by one nucleotide after each step. Depth of coverage along FlcDNA1028 obtained without read trimming (black) and with read trimming (red). Median depths of coverage for the gene obtained without read trimming or with read trimming are represented, respectively, by black and red horizontal lines. [file gb-2011-12-9-r88-S1.PDF]

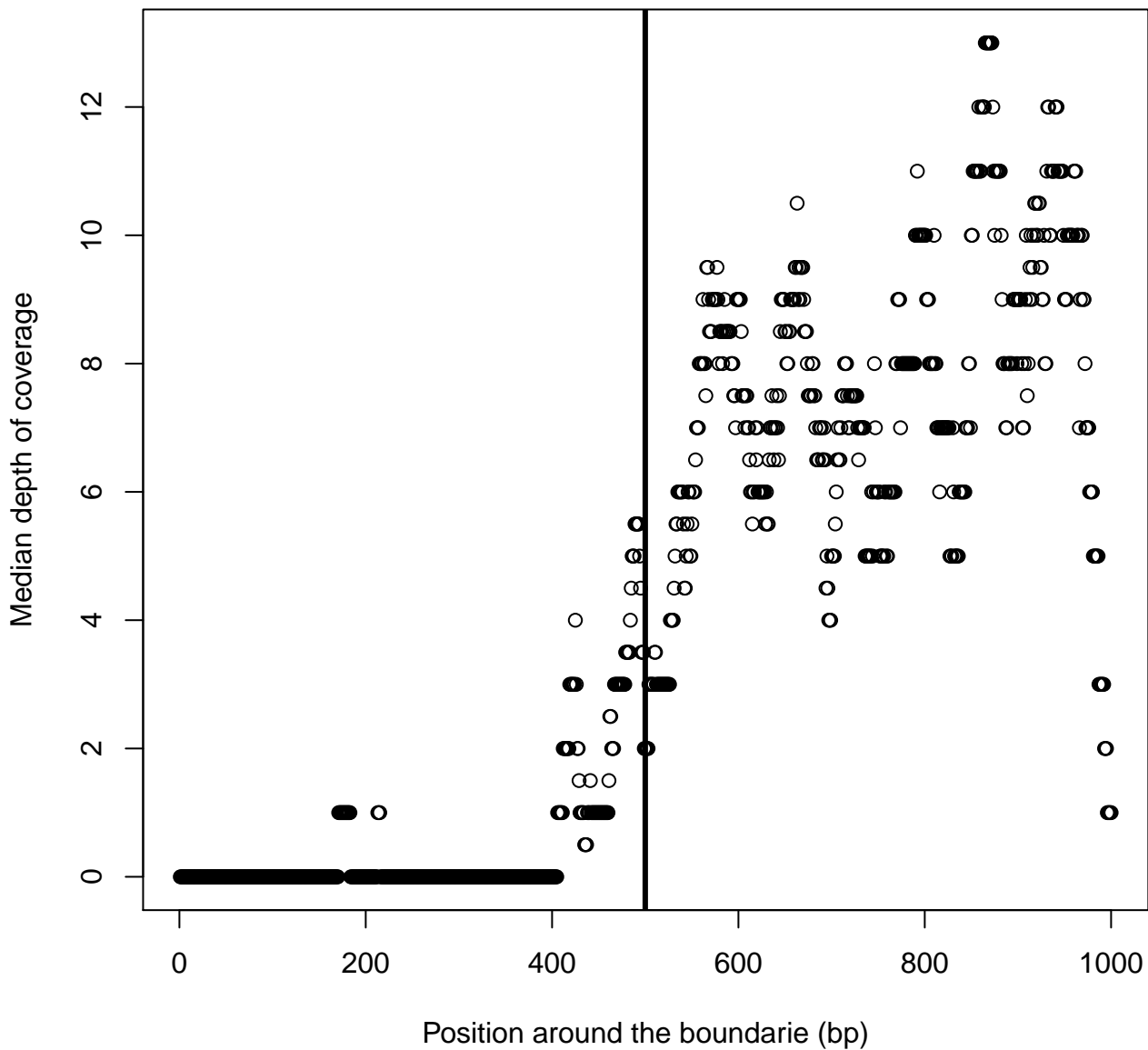

Supplement: Additional file 3 — Median depth of coverage per base around GPC locus target boundaries. A set of 17 boundaries from the GPC locus having a depth of coverage less than 61× was selected. The average MDC was calculated for 17 off-target/on-target boundaries, including 500 bp upstream and downstream sequences. [file gb-2011-12-9-r88-S3.PDF]

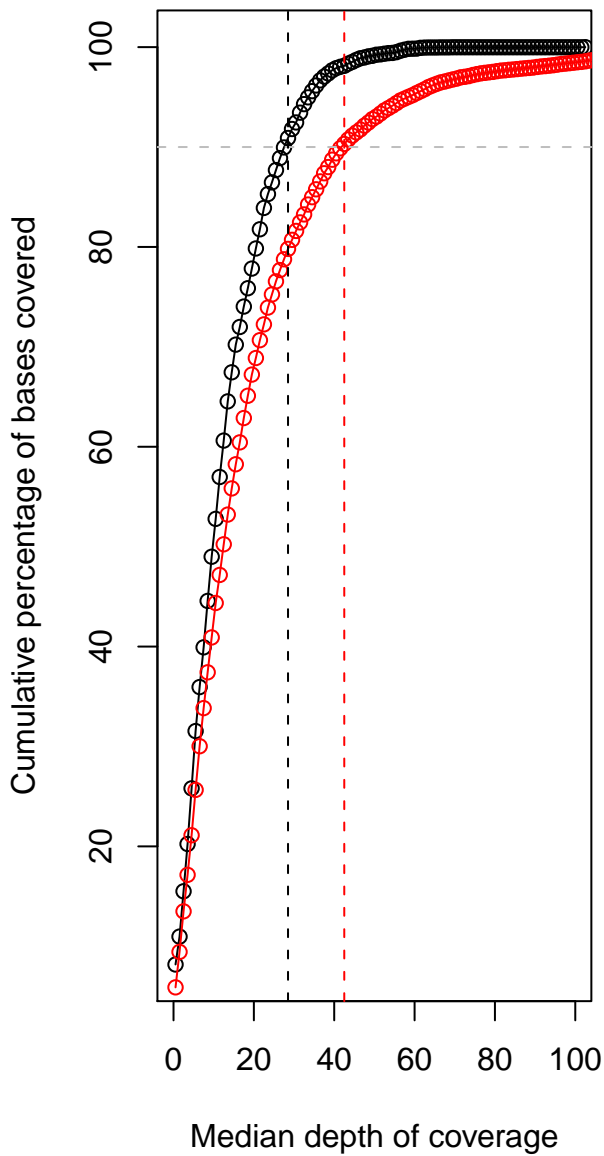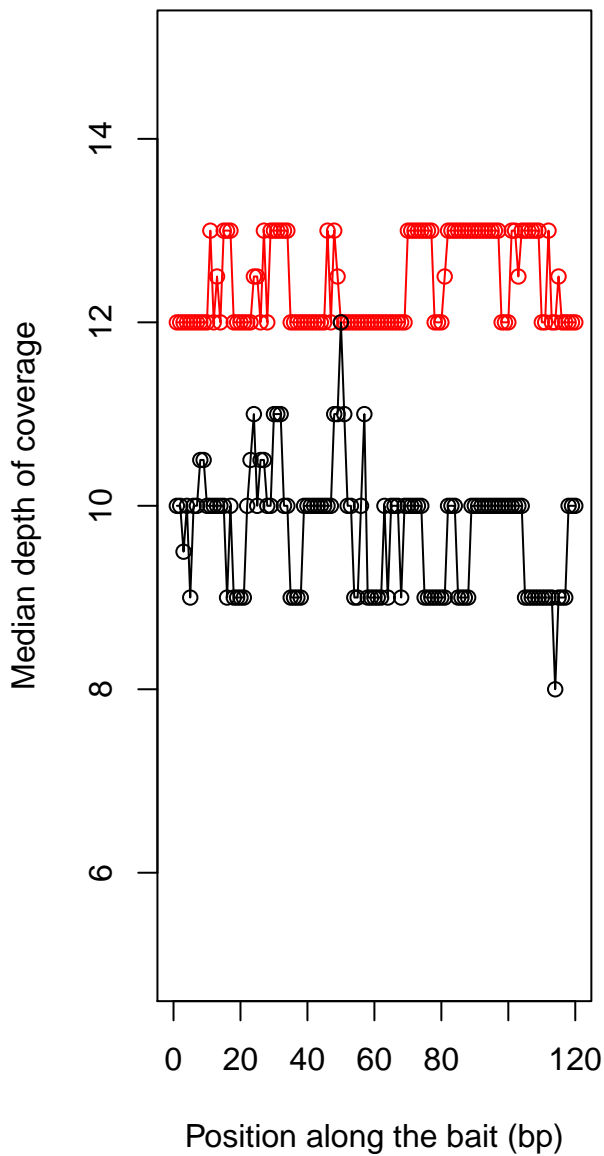

Supplement: Additional file 5 — Capture efficiency for 1× and 2× tiled regions of the GPC locus. (a) The cumulative distribution of MDC for 1× tiled (black lines) and 2× tiled regions (red lines). (b) The MDC per base along the bait for 1× tiled (black lines) and 2× tiled targets (red lines). [file gb-2011-12-9-r88-S5.PDF]

Number of mismatches

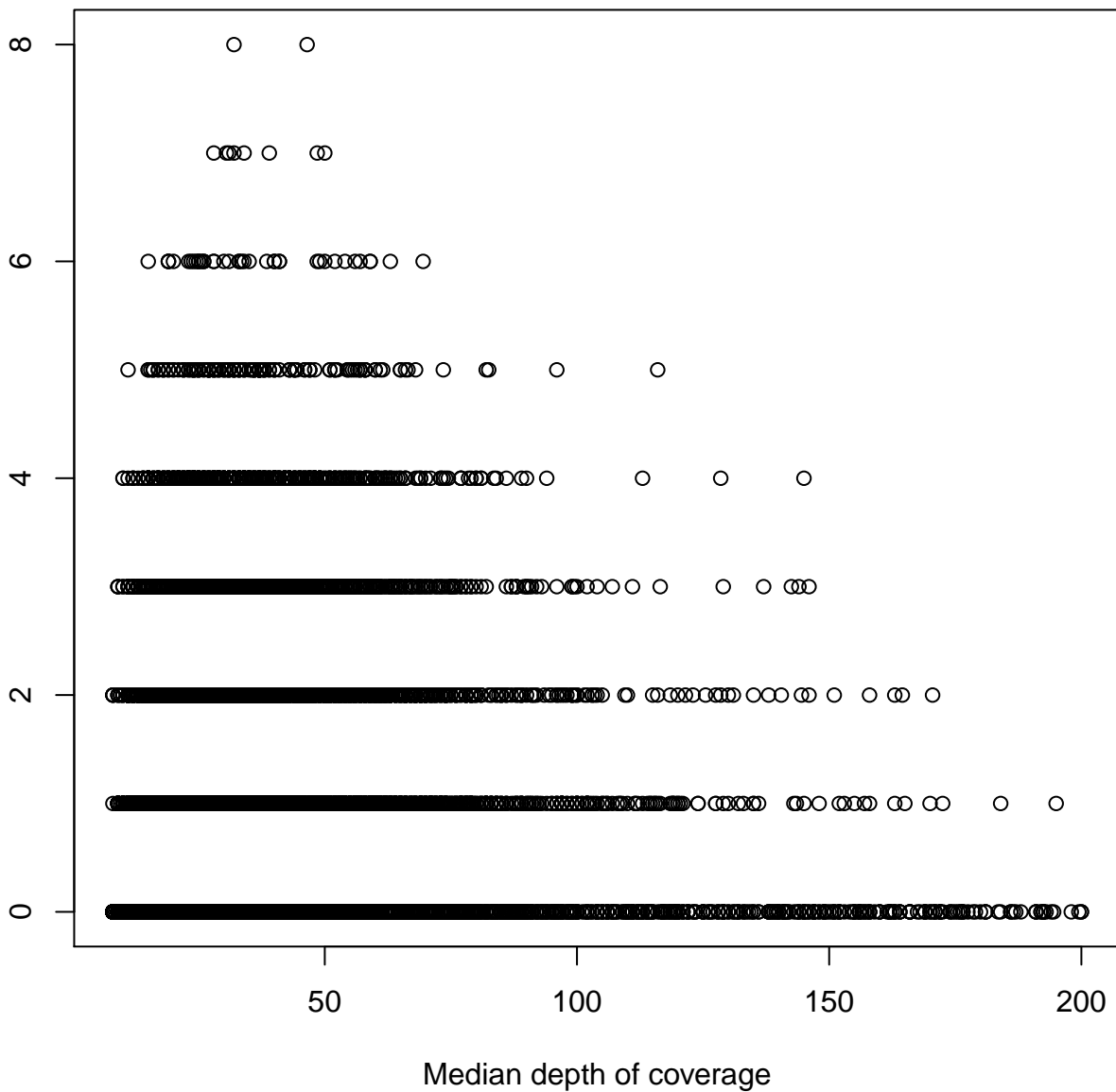

Supplement: Additional file 9 — Impact of mismatches between captured DNA and bait sequences on the median depth of coverage. The number of mismatches (includes SNPs and GSSs discovered between Ld and Td) between the captured DNA and bait sequences were plotted against the median depth of target coverage obtained for a region covered by bait. [file gb-2011-12-9-r88-S9.PDF]
