# Supplementary material for: Targeted analysis of nucleotide and copy number variation by exon capture in allotetraploid wheat genome
Source: Genome Biol. 2011 Sep 14;12(9):R88. doi: 10.1186/gb-2011-12-9-r88 (PMC3308051; doi:10.1186/gb-2011-12-9-r88)
Supplement: Additional file 4 — Influence of bait properties on capture efficiency. MDC was calculated for 47,874 2× tiled baits. All baits with a MDC above 200 were removed from the analysis. MDC was plotted against different bait parameters values: GC, bait GC content; k-mers, median frequency of the bait sequence in the Chinese spring genome; PMFE, probe minimum folding energy; PHFE, probe hybridization free energy; HS, bait Hairpin score; DS, bait Dimer score; Tm1 and Tm2, melting temperatures 1 and 2. Details of parameter estimations are provided in the Materials and methods. Red curves represent the median of MDC per value of a parameter. [file gb-2011-12-9-r88-S4.PDF]

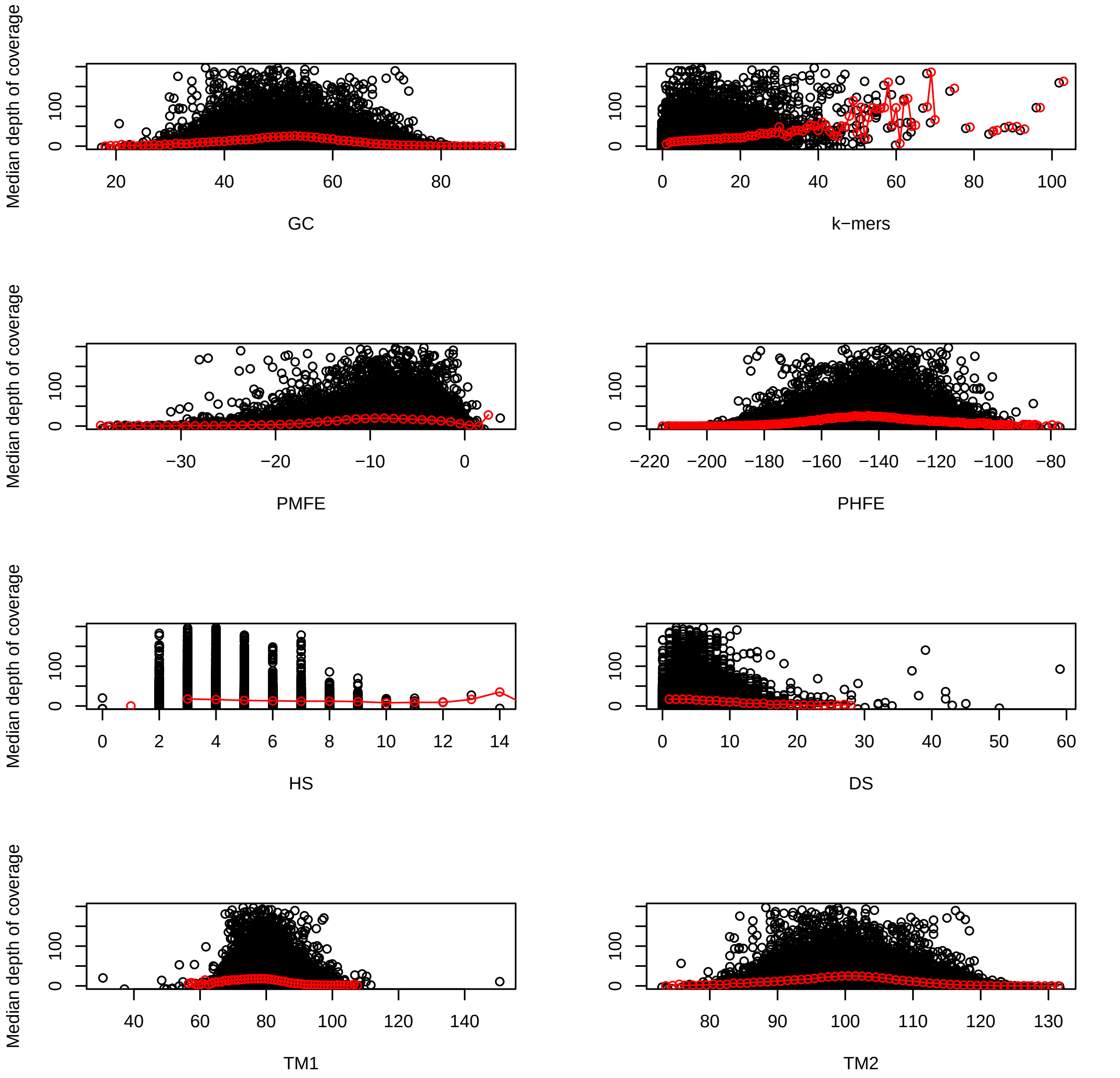

Median depth of coverage
GC
k−mers
PMFE
PHFE
HS
DS
TM1
TM2
